# A non-calorimetric approach for investigating the moisture-induced ageing of a pyrotechnic delay material using spectroscopies

**DOI:** 10.1038/s41598-019-51667-y

**Published:** 2019-10-23

**Authors:** Ji-Hoon Ryu, Jun-Ho Yang, Jack J. Yoh

**Affiliations:** Department of Mechanical and Aerospace Engineering, Seoul National University, Seoul, 08826 South Korea

**Keywords:** Energy science and technology, Materials science

## Abstract

The degradation of thermal properties due to ageing such as burning rate and exothermic heat release are unsolved issues faced during a long-term storage of the pyrotechnic substances. Accordingly, we employed various non-calorimetric methods to investigate the thermal performance of pyrotechnic delay, which is exposed to various moisture-rich conditions at extended durations. The chemical and physical changes in the compositions of a pyrotechnic delay comprised of metal fuel (Zr-Ni alloy) and oxidants (KClO_4_, BaCrO_4_) are analysed for four different relative humidity levels using X-ray photoelectron spectroscopy, X-ray diffraction, scanning electron microscope and laser-induced breakdown spectroscopy. The calculations using the NASA Chemical Equilibrium with Applications (CEA) software indicated that the heat of reaction for the components stored under the moisture-rich conditions is reduced by more than 50%. Unlike the conventional calorimetric analysis, the present non-calorimetric approach provided the compositional changes as well as the cause and effect of the relevant ageing process of pyrotechnic delay.

## Introduction

Owing to their designed exothermic characteristics, the pyrotechnic materials for providing the high energy impulse through chemical reactions of various time delays have been commonly adapted in the applications of military and industrial uses. The energy composites composed of fuels and oxidants have been designed to exhibit required performances from sequential heat generation with increasing temperature over a wide range. Highly reactive metal powders including zirconium (Zr), titanium (Ti), boron (B) and aluminium (Al), are used as fuel in the pyrotechnic mixture owing to their abundance and extremely explosive nature in powder form. In oxygen-rich environments, these metals undergo rapid combustion, transforming into thermally stable and chemically inert metal oxides. Suitable oxidants can improve the combustion efficiency of such systems by releasing more energy during the decomposition under a continuous oxygen supply. As a result, oxidant mixing has been proposed as an effective way to improve the energy release of the metal fuels in a solid powder form^1–5^.

However, when these mixed materials are stored for a long period of time, they undergo a process known as ageing, leading to the significant degradation of activation energy and the intended heat of combustion^6,7^. Also, the rate of reaction is decreased, inhibiting the impulsive energy release originally intended. In addition, the ageing due to oxidation is associated with pre-consumption of the reactants, also resulting in a decreased energy release. This leads to a misfire or a combustion failure owing to a combustion instability. In the pyrotechnic industry, the ageing of an ignition material is highly correlated with increasing budget owing to reduced reliability and shortened life span. Therefore, to ensure long-term stability, it is important to understand the ageing mechanisms that alter or inhibit the intended performance of material. Both naturally and artificially aged samples are used for such investigations, depending on the purpose of the analysis. Here, artificially accelerated ageing involves intentional degradation designed to determine the effects of certain conditions on a substance. By observing the effects of artificial stress over a short period of time, one can predict the shelf life of substances in advance^8,9^.

A variety of ageing studies have recently been conducted on metal fuel/oxidant pyrotechnic materials^10–18^. Here, moisture is a factor that can cause ageing of pyrotechnic materials. Exposure to moisture is an inevitable threat to a pyrotechnic substance during the pre-packaging stage or typical storage periods. There have been several studies focusing on the effects of moisture on pyrotechnic mixtures^13–18^. Lee analysed aged BKNO_3_ (boron/potassium nitride) at 71 °C at 50% relative humidity (RH) using differential scanning calorimetry (DSC), thermogravimetric analysis (TGA), Fourier-transform infrared spectroscopy (FTIR), X-ray photoelectron spectroscopy (XPS) and transmission electron microscopy-energy dispersive spectroscopy (TEM-EDS)^13^. As a result, the researchers revealed that high humidity is a potential cause of energy loss due to the formation of oxides on the boron surface. Kim *et al*. demonstrated that pure Zr/KClO_4_ is more sensitive to the production of oxides compared to other materials by measuring flame temperature as exposure to an oxygen source (H_2_O or O_2_ atmosphere) increased^14^. Furthermore, it was confirmed that the addition of oxygen causes generation of H, OH radicals from KO, and ClO radicals and H_2_O from the oxidising agent. Babar *et al*. confirmed that micro-cracks form on solid Mg/NaNO_3_ surfaces at 70 °C and RH 70% using scanning electron microscope (SEM) and confirmed the oxidation of Mg by X-ray diffraction (XRD)^15^. Oh *et al*. carried out the kinetic analysis of Zr/KClO_4_ with DSC and XPS at the degree of ageing according to the relative humidity (RH) level and estimated the shelf life of the material with the increase of ZrO_2_^16^. Furthermore, Wang *et al*. derived the critical temperatures for the oxidisers in pyrotechnic substances at differing degrees of moisture-induced ageing and revealed the adverse effect of moisture on the thermal stabilities of the substances^17^. In addition, Muhammad *et al*. demonstrated that the moisture contents of ammonium-perchlorate-based composite propellants causes degradation of their mechanical properties, such as tensile strength and elongation^18^. Thus, these previous studies demonstrate that moisture is a major cause of fuel oxidation of pyrotechnic materials, but do not elaborate on the ageing mechanism. Furthermore, spectroscopy is utilized in a limited way only to support the calorimetry results.

This study aims to implement the application of the spectroscopic approaches, unlike the conventional calorimetric approaches, to the study of pyrotechnic materials as a means to elucidate the moisture-induced ageing mechanisms and to evaluate the thermal performance. The samples, composed of metal fuel (Zr-Ni alloy) and oxidants (BaCrO_4_, KClO_4_), were prepared under moisture-induced ageing conditions according to different exposure levels. Four spectroscopic techniques were used to investigate the ageing in the pyrotechnic delay material, and one calorimetric analysis was performed to verify their results. XPS and XRD revealed the chemical changes in each component and their causes upon ageing. SEM analyses showed the physical changes, such as evidence of hydrogen embrittlement of the metal fuels due to excessive H_2_O. In the case of laser-induced breakdown spectroscopy (LIBS), the oxidation levels of the metal fuel in the materials was effectively determined. The compositions of each component as revealed by the spectroscopic analyses were used to predict the material’s behaviour in terms of heat of reaction using NASA’s Chemical Equilibrium with Application (CEA) software. A comparison of the results with those obtained by DSC was conducted to ensure the reliability of the spectroscopic results. By focusing on changes in each composition due to ageing, spectroscopic analysis provides important guidelines that go beyond calorimetric analysis in the thermal behaviour and causes. Thus, this work presents a methodology to enrich our understanding of ageing mechanisms using non-calorimetric approaches to determine the underlying causes of ageing.

## Results

### XPS and XRD results

#### *Effect of moisture on KClO*_4_

**Figure 1:**
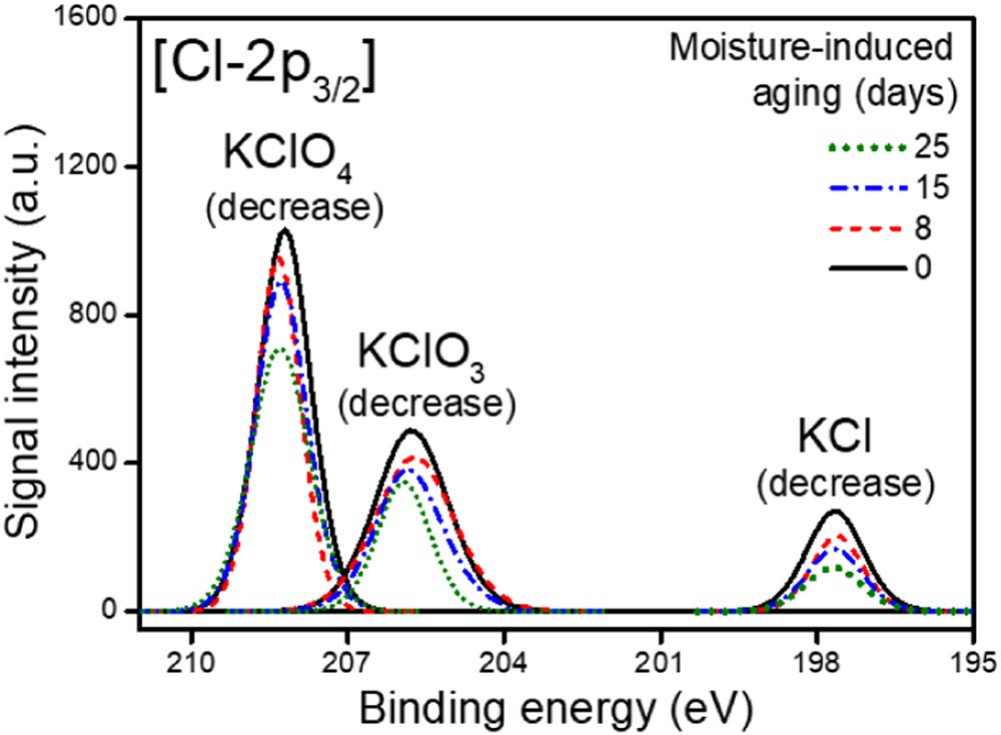
Cl2p_3/2_ XPS peaks for a pyrotechnic delay material at different levels of moisture-induced ageing, indicating that long-term exposure in moisture-rich conditions tends to reduce the contents of KClO_4_ (208.30 eV), KClO_3_ (205.90 eV) and KCl (197.80 eV).

KClO_4_ can generate a large quantity of heat upon reacting with Zr-Ni fuel at around 500 °C^19^. However, several researchers have reported that the weak Cl-O bonds can be broken at room temperature, resulting in the reduction of KClO_4_ with the release of oxygen atoms^20^. Also, other study has confirmed that KClO_4_ in a pyrotechnic substance can be reduced toward the decomposition of O, to form KClO_3_ or KCl^16,21^. In the present study, XPS results demonstrated how KClO_4_ changes under excessive moisture conditions. Figure 1 shows the sequential behaviour of KClO_4_ and its reaction products in the long-term exposed pyrotechnic delay material under the excessive moisture conditions. The Cl2p_3/2_ XPS spectra of the samples exhibit visible features at 208.30 eV, 205.90 eV and 197.80 eV. In the pristine sample or the unaged sample, the presence of KClO_3_ and KCl is confirmed, which indicates that KClO_4_ is unstable due to weak Cl-O bonds and decomposes at room temperature. In addition, there may be manufacturing defects in the initial potassium perchlorate used during the synthesis of the pyrotechnic mixture^22^.

The KClO_x_ (0 ≤ x < 4) content gradually decreases depending on the duration of moisture exposure. This is due to the high solubility of KClO_x_ in water under the experimental conditions. Hori *et al*. reported that ClO_4_^−^ dissolved from KClO_4_ can be converted to Cl^−^ by oxidising Fe and FeO to Fe_2_O_3_^21^. In this study, ClO_4_^−^ was effectively dissolved on the surface of the Zr-Ni alloy due to the excessive moisture level and reduced to ClO_x_^−^ (0 ≤ x < 4) upon reacting with the highly reactive Zr, Ni^23^. Upon dehydration after moisture-induced ageing, the dissolved ClO_x_^−^ does not readily convert back to conventional solid-phase KClO_x_, leading to a decrease in the total KClO_x_ content. Furthermore, in ageing period longer than the experimental conditions, most KClO_4_ is likely to decompose into KClO_x_ (0 ≤ x < 4)^21^. Thus, the moisture absorbed by the pyrotechnic delay material plays an important role in dissolving KClO_4_ and allowing its reduction on the surface of the Zr-Ni alloy, converting it to K^+^ and ClO_x_^−^ (0 ≤ x < 4).

#### Effect of moisture on BaCrO_4_

**Figure 2:**
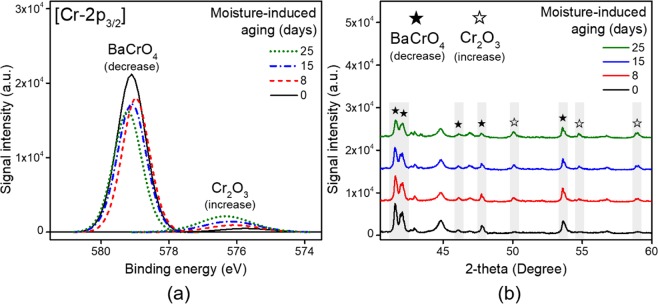
Spectral results showing the changes in BaCrO_4_-related components due to moisture-induced ageing. (**a**) XPS and (**b**) XRD results. Both spectroscopic analyses reveal the decomposition of BaCrO_4_ to Cr_2_O_3_ upon moisture-induced ageing.

BaCrO_4_ is regarded as an essential component of a pyrotechnic delay material because it is useful as a burn rate modifier and a heat source^24^. In addition, since BaCrO_4_ is an important component, its stability is essential for normal operation of the pyrotechnic delay. Figure 2 demonstrates the effects of moisture on BaCrO_4_ through XPS and XRD results. As shown in Fig. 2(a), a signal for Cr_2_O_3_ at 576.70 eV appears with a concomitant decrease in the signal for BaCrO_4_ at 578.80–579.10 eV as ageing progresses. The change in Cr_2_O_3_ is not large, but it is clearly observed. In the XRD patterns shown in Fig. 2(b), the major peaks for BaCrO_4_ (2θ = 41.5°, 42.1°, 46.1°, 47.8° and 53.6°) and Cr_2_O_3_ (2θ = 50.1°, 54.5° and 58.9°), corresponding to JCPDS files 15-0376 and 38-1479, respectively, are clearly observed^25,26^. There is a gradual change in the BaCrO_4_ and Cr_2_O_3_ signals depending on moisture exposure level. Because these two components are the only ones associated with Cr, it is apparent that the BaCrO_4_ is reduced to Cr_2_O_3_ under excessive moisture conditions.

In general, BaCrO_4_ is ionised to Ba^2+^ and CrO_4_^2−^ in acidic conditions, despite its low solubility in water, and reacts as follows:

$$2{{\rm{CrO}}}_{{\rm{4}}\,{\rm{aq}}}^{2-}+2{{\rm{H}}}_{{\rm{aq}}}^{+}\,\mathop{\longleftrightarrow }\limits^{{H}^{+}}\,{{\rm{Cr}}}_{2}{{\rm{O}}}_{{\rm{7}}\,{\rm{aq}}}^{2-}+{{\rm{H}}}_{2}{\rm{O}}({\rm{l}})$$

$${{\rm{Cr}}}_{2}{{\rm{O}}}_{{\rm{7}}\,{\rm{aq}}}^{2-}+14{{\rm{H}}}_{{\rm{aq}}}^{+}+6{{\rm{e}}}^{-}\to 2{{\rm{Cr}}}^{3+}+7{{\rm{H}}}_{{\rm{2}}}{\rm{O}}\,\mathop{\longrightarrow }\limits^{dehydration\,\& \,oxidation}\,{{\rm{Cr}}}_{2}{{\rm{O}}}_{3}({\rm{s}})$$

here, the influx of hydrogen ions is essential for the reduction of the material. These hydrogen ions may be derived from the accelerated ageing process or H_2_CO_3_ generated from CO_2_ dissolved in the water, in accordance with Henry’s law^27^. However, these interpretations are speculative and require further research.

Thereafter, the CrO_4_^2−^ coexists with Cr_2_O_7_^2−^ according to Le Chatelier’s principle, and the valence of some of the Cr is decreased from 6 to 3. Cr(III) is converted to chromium(III) oxide (Cr_2_O_3_) upon dehydration and oxidation during the accelerated ageing process^27^. Thus, the decomposition of BaCrO_4_, which is ionised under humid-acidic conditions, into Ba and Cr_2_O_3_ is one of the main factors that significantly decreases the heat release of related pyrotechnic materials with time.

#### Effect of moisture on the metal fuel

**Figure 3:**
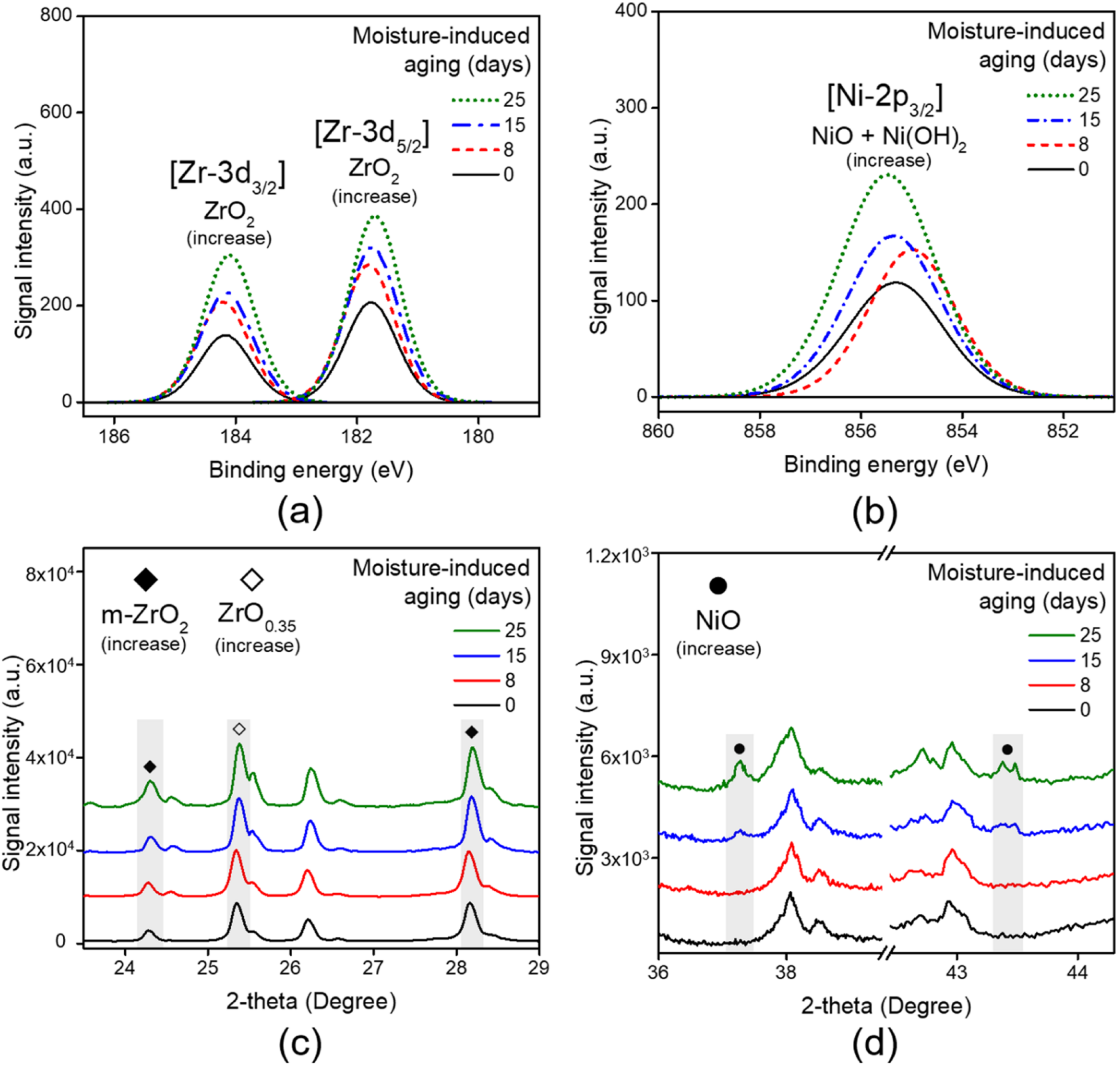
(**a**,**b**) XPS and (**c**,**d**) XRD analyses of the metal fuel (Zr-Ni alloy) in a pyrotechnic delay with regards to moisture exposure level. In both analyses, the signals for ZrO_2_, NiO and Ni(OH)_2_ increase with ageing. The results demonstrate that moisture is an important factor in the pre-oxidation of the metal fuel. The XRD patterns in (**c**) are offset by 9,500 a.u. for clarity. Those in (**d**) are offset by 1,500 a.u.

Figure 3 shows the XRD and XPS results for the metal fuel component with regards to the moisture exposure level. The chemical changes in the metal fuel are confirmed from the Zr3d and Ni2p peaks, as shown in Fig. 3(a,b). Pre-oxidation of Zr is evident in a pair of orbital doublets corresponding to Zr-3d_5/2_ (about 182.20 eV) and Zr-3d_3/2_ (about 184.00 eV), as shown in Fig. 3(a). In Fig. 3(b), the signal in the region of about 855.0 eV gradually increases with time. This signal appeared due to the mixture of NiO and Ni(OH)_2_ signals, indicating that ageing of nickel is being accelerated by the level of moisture exposure^28^. Figure 3(c,d) reveals the same tendency in the XRD results, showing the formation of oxides of Zr and Ni depending on the degree of moisture exposure. In Fig. 3(c), it is noticeable that the formation of the m-ZrO_2_ crystal phase (2θ = 24.2° and 28.2°, JCPDS #37-1484^29^) increases with ageing. It is also confirmed that an oxygen-deficient metal oxide is formed and coexists during the oxidation of Zr from the intense diffraction signal for ZrO_0.35_ at 25.4° (JCPDS #17-0385)^29^. Meanwhile, as shown in Fig. 3(d), NiO signals emerge at 37.3° and 43.3° after 8 days of ageing (JCPDS #47-1049)^30^.

The significant ZrO_2_ signals for the pristine sample result from the immediate formation of a thin oxide film due to the low electronegativity of the metal and the high electron affinity of oxygen^31^. Acting as a diffusion barrier, the oxide layer gradually thickens over time as the adsorbed oxygen slowly permeates into the metal^32^. In addition, the reaction is promoted in the presence of moisture and the oxidising agent^33^. As discussed earlier, the reaction of the metal with ClO_4_^−^ is a major factor in its oxidation. Furthermore, the ageing due to oxidation can be exacerbated by the increase in surface area upon the formation of cracks, as will be mentioned later. Conversely, unlike that of Zr, the oxidation of Ni is relatively independent of moisture level. Despite the high content of Ni, the low signal intensity for NiO in the XRD results results from the high corrosion resistance of the metal to the oxygen environment^34^. Or, it is due to the fact that the oxides of nickel can convert to hydroxides in humid ambient. Therefore, it is reasonable to assume that the increase in NiO signal with moisture exposure is due to an increase in surface area rather than the growth of the oxide layer. Thus, moisture can significantly accelerate the pre-oxidation of Zr-Ni alloys.

### SEM results

**Figure 4:**
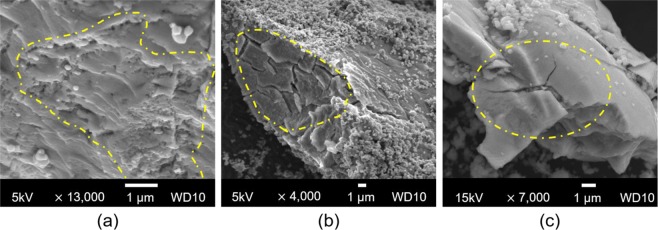
SEM images for 25-day-aged samples showing representative morphology defects visible on the Zr-Ni alloy. (**a**) Voids, (**b**) cracks in the crystal grain boundary, and (**c**) a split.

Figure 4 shows several types of severe defects on the surface of the Zr-Ni based alloy for the 25-day-aged samples. Unlike the pristine sample, the aged Zr-Ni alloy appears to have undergone hydrogen embrittlement. As mentioned in several studies, it is generally agreed that bulky H_2_O is a powerful agent for promoting hydrogenation to generate hydrogen-assisted cracking^35^. Highly reactive Zr-Ni alloys are subject to intrusion by H_2_O due to micro-cracks originating from micro-defects formed during transport and storage. In addition, Cl ions are gradually generated due to moisture-induced ageing, as discussed above, and these ions can cause pitting corrosion, which accelerates the formation of cracks in the Zr-Ni alloy^36^. Thereafter, the ingress of hydrogen from the adsorbed moisture into the metal occurs. Due to its high diffusivity, hydrogen diffuses to the grain boundaries along the lattice structure of the Zr-Ni alloy and degrades the grain cohesion. Moreover, since impurities in the alloy tend to separate from the grain boundaries due to their poor binding with the base material (Zr, Ni), hydrogen also tends to settle in the structural defects, such as dislocation and micro-voids, generated in this process^37^. Over time, hydrogen gas forms, resulting in severe splitting of the alloy due to rapid expansion. The cracks abate the surface energy of the alloy, thereby increasing the fuel surface area^38^. Consequently, the particle size gradually decreases with ageing. Solid propellants are made in powder form for effective reaction, but excessively small particles worsen the pre-oxidation of the metal due to their large surface area, leading to misfires or failures. In addition, decreasing the particle diameter of metal fuels due to cracking leads to a rapid burning rate, which will reduce the effectiveness of the pyrotechnic delay^39^. Consequently, the performance of fuel affected by moisture deviates from its intended design specifications.

### LIBS results

LIBS is a convenient plasma emission spectroscopic technique for high-sensitivity elemental analysis in real-time without pre-processing. LIBS allows qualitative and quantitative analyses by obtaining spectral information from the plasma emitted by laser irradiation over a local area^40^.

In a previous study, we measured the ZrO_2_ content of a pyrotechnic material (Zr/Fe_2_O_3_) through laser ablation by detecting the Zr-O bond emissions during the plasma cooling process^12^. Here, the area under the curve (AUC) method was used for quantitative analysis of the ZrO signal^41^. Furthermore, in order to determine the ZrO_2_ content of the aged samples, a calibration curve was constructed from the ZrO signal using non-aged samples spiked with ZrO_2_. In the present study, we used the same approach to determine Zr oxidation levels upon moisture-induced ageing.

**Figure 5:**
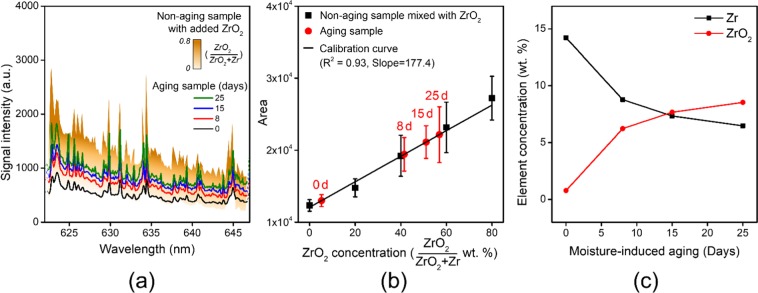
Determination of Zr oxidation level from the ZrO signal in LIBS according to moisture-induced ageing. (**a**) Detection of the ZrO molecular signal for non-aged and aged samples. Here, the non-aged samples were prepared by varying the value of x in Table 3 from 0 to 0.8. (**b**) Construction of a calibration curve from AUC results. (**c**) Determination of the oxidation level of Zr upon ageing. These results indicate that exposure to moisture is fatal to the fuel component in a pyrotechnic delay material.

Figure 5 illustrates the experimental procedure used to determine the level of Zr oxidation through LIBS. First, we obtained the LIBS spectra for four moisture-induced ageing samples and five non-aged samples spiked with ZrO_2_, as shown in Fig. 5(a). In the case of the non-aged sample, the change in the signal upon the increase in ZrO_2_ content is expressed in the form of a contrast. The ZrO molecular emission signal is clearly observed at 623–634 and 634–647 nm, which are ZrO α (1,0) bands for a b^3^Φ − a^3^Δ system consisting of b^3^Φ_4_ − a^3^Δ_3_ and b^3^Φ_3_ − a^3^Δ_2_ subsystems^42^. The molecular signals related to the electronic, vibrational, and rotational transitions of the Zr-O bonds gradually increase with ZrO_2_ content. The signals for the aged samples increase with moisture exposure level, as expected. Then, as shown in Fig. 5(b), a calibration curve for ZrO_2_ concentration was constructed from the AUC result for the ZrO signal of the spiked non-aged samples. The linear fit follows the equation y = 177.4 × + 12056.7 (R^2^ = 0.97). Thereafter, the AUC results for the aged samples were matched with the regression line to confirm the increase in ZrO_2_ content with ageing. All data processing was performed using OriginPro software (OriginLab, OriginPro 8.5.1, USA). Here, the spectrum of a pure paraffin binder was used as the baseline owing to its inactivity in LIBS. Finally, the change in Zr and ZrO_2_ contents according to moisture exposure level can be seen in Fig. 5(c). The results show that 5.2%, 41.0%, 51.1% and 56.9% of the Zr are oxidised to ZrO_2_ upon moisture-induced ageing for 0, 8, 15 and 25 days, respectively. Thus, high moisture conditions gave rise to the pre-reaction of the Zr metal fuel with oxygen.

### Estimation of thermal performance through non-calorimetric analyses

Table 1 lists the elemental contents in a pyrotechnic delay material subjected to moisture-induced ageing obtained from the spectral results. The compositions of the oxidiser-related constituents were determined through quantitative XPS analyses. In addition, the concentrations of the Zr-based substances were determined by the LIBS analyses. The oxidation of Ni was assumed to be at the same level as that of Zr. Since Rareox #14 is barely present, its analysis was excluded for the purpose of this study. As expected, oxidation of the fuel and decomposition of the oxidiser became more evident as the ageing progressed.

**Table 1:** Compositional changes of a pyrotechnic delay material according to moisture-induced ageing level.

Ageing condition		0 days	8 days	15 days	25 days
Fuel	Zr	14.22	8.85	7.33	6.47
	ZrO_2_	0.78	6.15	7.67	8.53
	Ni	16.11	10.03	8.31	7.33
	NiO + Ni(OH)_2_	0.89	6.97	8.69	9.67
Oxidiser	BaCrO_4_	52.27	50.34	48.92	46.84
	Cr_2_O_3_	0.72	2.66	4.08	6.16
	KClO_4_	8.07	7.16	6.56	5.19
	KClO_3_	3.83	3.12	2.82	2.53
	KCl	2.11	1.53	1.23	0.86
Enthalpy of reaction (ΔH) [J/g]		−1,998.07	−1,207.14	−995.33	−908.74

The heat of reaction (ΔH) is an effective indicator for evaluating thermal performance. In this study, we estimated the heats of reaction for a pyrotechnic delay material on the basis of spectral results given in Table 1 using the CEA software. Here, the reaction between Zr-Ni alloy, KClO_4_, and KClO_3_ was considered. The formation enthalpy of the elements for calculations were obtained from Chase *et al*.^43^ and the Cheetah program^44^. Only the total mass of BaCrO_4_ in the combustion reaction is considered because it acts independently from the other elements and notably as a key factor in producing the delay time.

**Figure 6:**
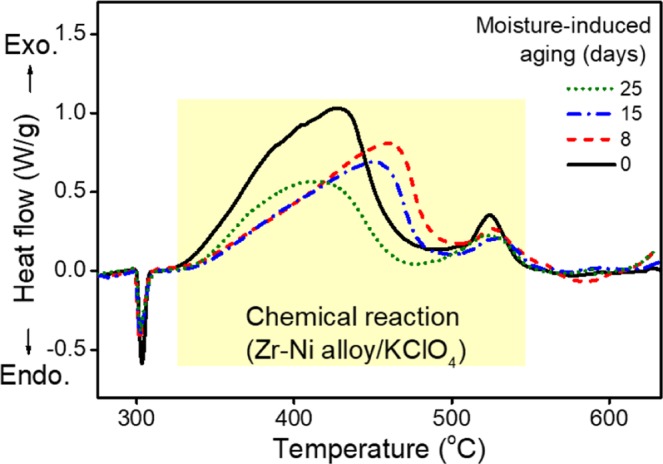
DSC curves for a pyrotechnic delay according to moisture-induced ageing. An exothermic peak due to the reaction between fuel and oxidant is observed at 325–550 °C.

We also validated the feasibility of the present spectrometry-based analyses by the comparison with the results obtained using DSC, a best known calorimetric analysis. Figure 6 shows the DSC results for the moisture-induced ageing of the pyrotechnic delay material. The DSC curve shows a sharp endothermic peak at ca. 300 °C representing a phase change for KClO_4_, allowing quantitative analysis of KClO_4_. Furthermore, the two gentle peaks in the range 325–550 °C are characteristic of the inherent exothermic reaction of Zr-Ni alloys, KClO_4_, and KClO_3_. Heat flow is most dramatic in the pristine sample and progressively smooths with ageing. For the DSC results, the heat of reaction is calculated by constructing a tangential-sigmoidal baseline for the exothermic peak in the reaction temperature range for each sample. Dynamic calculations for combustion of the pyrotechnic delay were performed using AKTS-thermokinetics software^10^.

Table 2 shows changes in the heats of reaction for the pyrotechnic delay as ageing progresses as derived by two different analytical methods. Decreases in the heats of reaction over time are evident by both methods. However, the difference exists between the approaches for the following reason. First, the DSC experiments were performed under non-ideal conditions and their results are subject to incomplete combustion of KClO_4_. The decomposition of KClO_4_ proceeds up to ca. 1,000 °C, which is within the temperature range considered for CEA analysis but higher than that of DSC analysis^45^. Furthermore, BaCrO_4_ lowers the heat of reaction by absorbing the energy generated from the chemical reaction due to its high decomposition temperature, which exceeds the operating temperature limit of the DSC device.

**Table 2:** Comparison of the heats of reaction (ΔH) values and heat degradation ratios according to moisture-induced ageing levels as obtained from non-calorimetric (CEA) and calorimetric analyses (DSC).

Moisture-induced ageing condition [days]	Non-calorimetric results from CEA		Calorimetric results from DSC	
	Enthalpy of reaction [J/g]	Heat degradation ratio (%)	Enthalpy of reaction [J/g]	Heat degradation ratio (%)
0	−1,998.07	100.00	684.69	100.00
8	−1,207.14	60.41	445.39	65.04
15	−995.33	49.81	373.79	54.59
25	−908.74	45.48	340.30	49.70

**Figure 7:**
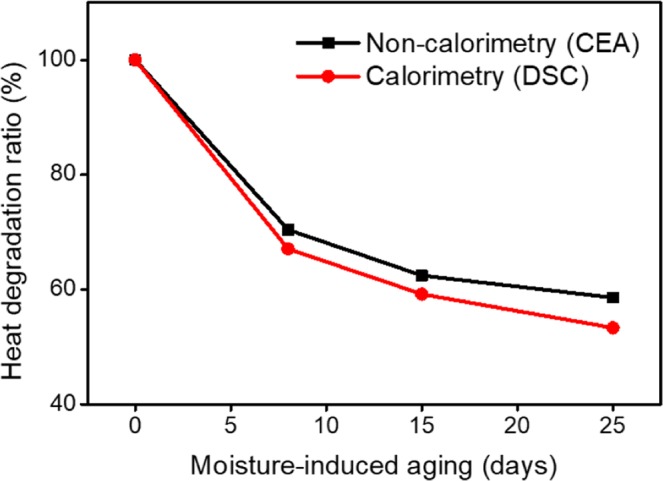
Comparison of the heat degradation ratios (ΔH_aged_/ΔH_pristine_) obtained using non-calorimetric (CEA) and calorimetric analyses (DSC). The trends in thermal performance as obtained by both approaches are similar.

Figure 7 shows the heat degradation ratios according to the ageing period calculated from CEA and DSC analyses. The heat degradation ratio is defined as ΔH_aged_/ΔH_pristine_ and allows the aged samples to be analysed in terms of the enthalpy change relative to the pristine samples. The decreasing trend in the heats of reaction revealed through CEA calculations is similar to that from the DSC results. The enthalpies of the aged substances as compared to those of the pristine samples are decreased to 60.41%, 49.81% and 45.48% according to the CEA results and to 65.04%, 54.59% and 49.70% according to the DSC results. From the similar results obtained by the two methods, we can conclude that changes in the low-temperature chemical reactions (below 650 °C) due to moisture are the predominant cause of deterioration in the thermal performances of a pyrotechnic delay material. Furthermore, our results indicate that the thermal performances of a pyrotechnic delay material can be predicted by spectroscopic analyses as an alternative to conventional thermal analyses and calorimetry.

## Discussion

In this study, we used spectroscopic approaches to investigate the variations in composition and thermal performance caused by moisture-induced ageing for a pyrotechnic delay material, which was prepared and aged at 71 °C and 70% RH for 0, 8, 15 and 25 days. Using XPS and XRD, we noticed a decrease in the mass of the oxidant and an increase in pre-oxidisation of the metal fuel with ageing. Because KClO_x_ is highly soluble in water and has weak Cl-O bonds, it is gradually dissolved and decomposed under the moisture-rich conditions, leading to a decrease in its total content. In addition, though it is difficult to identify the source of the hydrogen ions, they play an important role in ionising BaCrO_4_ and thus reducing CrO_4_ to Cr_2_O_3_. Also, SEM analyses confirmed that the formation and growth of metal oxide layers is promoted by the hydrogen-assisted cracking owing to the hydrogen atoms generated under high-moisture conditions. Furthermore, Cl ions promote the formation of micro-cracks on the metal surface through pitting corrosion. LIBS analyses further revealed that the fuel is oxidised by 5.2%, 41.0%, 51.1% and 56.9%, depending on the duration of moisture-induced ageing. Finally, the composition of the material was estimated based on the results of quantitative XPS and LIBS analyses. Through the spectroscopic analysis by using NASA CEA program, the heat of reaction according to the degree of ageing was decreased by ca. 39.59%, 50.19% and 54.52% compared to the pristine sample, which is similar to the result of conventional DSC. The novel non-calorimetric method is a powerful tool to simultaneously understand the changes in thermal performance and its mechanism as the ageing of the pyrotechnic delay progresses. Overall, for the pyrotechnic delay material, moisture-induced ageing results in a decline in oxidant content and the premature onset of fuel oxidation, resulting in deviations from intended performance.

## Methods

### Materials

Table 3 shows the basic compositions of the pyrotechnic delay material used in this study. Energy release is provided through the exothermic reaction of Zr-Ni alloy and KClO_4_. BaCrO_4_ is added to influence the delay time. Ni is added to Zr in the fuel due to its slow and reliable burning characteristics. A small amount of Rareox #14 provides mechanical binding between the fuel and oxidant. For each type of sample, a pristine sample without any chemical pre-processing was considered as a reference sample for the analyses. The samples were then subjected to moisture-induced ageing for 8, 15, or 25 days at 71 °C, and 70% RH. Based on the van’t Hoff equation, the accelerated ageing technique used for the high-humidity conditions used in this study can accurately reproduce the natural long-term ageing^46^. In order to construct a calibration curve for the quantitative LIBS analyses, non-aged samples spiked with ZrO_2_ were prepared, as shown in Table 3.

**Table 3:** Composition of the pyrotechnic delay material used in this study.

Component	Concentration (%)
BaCrO_4_	53
Zr	15 x
ZrO_2_	15 (1−x)
Ni	17
KClO_4_	14
Rareox #14	1

Note: x indicates Zr/(Zr + ZrO_2_) ratio (0 < x < 1.0) used for LIBS samples.

### Non-calorimetric methods

#### XPS

XPS analyses were performed using an AXIS SUPRA instrument (Kratos Analytical Ltd., UK). The spectrometer uses an automated monochromatic aluminium Kα X-ray source (hν = 1486.6 eV) with an ultimate energy resolution of ≤0.48 eV. The XPS spectra were collected using a 180° hemispherical electron energy analyser (WX-600) consisting of over 100 individual data channels. The energy positions of the spectra were calibrated with reference to the Ag3d_5/2_ level of clean silver at a pass energy of 20 eV. The analyte was delicately manipulated in terms of x, y and z directions and θ and Φ rotations in an ultra-high vacuum chamber. The low-pressure chambers for sample analysis and load-lock were set at 5 × 10^−10^ and 5 × 10^−8^ torr, respectively. The sources of binding energy were obtained from the NIST database and the related literature.

#### XRD

The XRD experiments were conducted using a high-resolution X-ray diffractometer (HR-XRD, Rigaku SmartLab, Japan). The diffractometer was operated at 9 kW with Cu Kα radiation at 1.541Å. The spectra were recorded at a scan rate of 0.5 °/min between 15° and 90° using a freely movable image detector (Hypix-3000) located on the top of the scanner (scan step 0.02 °/min). The XRD patterns were interpreted using Rigaku PDXL application software based on ICDD’s PDF-2 database.

#### SEM

The surface morphology and microstructure of the Zr alloy after 25 days of moisture-induced ageing were analysed by field-emission scanning electron microscope (FE-SEM, JSM-7800, Japan) at an acceleration voltage of 30 kV under vacuum conditions. Surface observation of the sample was accomplished by obtaining a high-resolution image with a very high signal-to-noise ratio using gentle beam super-high-resolution mode. The images are provided by integrating signals from four detectors, i.e., an upper electron detector and an upper secondary electron detector to detect electron energy, a backscattered electron detector to observe channelling contrast, and a lower electron detector to acquire information on surface roughness from the illumination effect. In addition, the SEM experiment was performed by adjusting the sample orientation from **−**5° to 70° on a xyz stage.

#### LIBS

Quantitative LIBS studies were performed using a 1064 nm Nd:YAG laser source (RT250-Ec, Applied Spectra Inc.) with 20 mJ energy and a 5-ns pulse duration. A somewhat converged laser from a beam expander was passed through a 15-times objective lens (LMM-15X-P01, Thorlabs) to provide a more sophisticated, chromatic-aberration-free energy source. In order to obtain a signal for the Zr-O bonds generated in the plasma cooling process, a laser-ablated sample subjected to laser irradiation at 2.54 × 10^12^ W/m^2^ over an average 100 um spot size was measured with a high-resolution 6-channel ICCD in the wavelength range 198~1050 nm. The generated plasma is collected through an optic fiber located at a 150 mm distance at 45°, and the detecting system was operated at a carefully chosen gate width (1.05 ms) and gate delay time (1.0 μs). For LIBS analyses, spectra were obtained by averaging approximately 100 pulses irradiated on the sample surface. In addition, the sample was pelletized at a pressure of 10 tonnes for a dwell time of 150 s and a release time of 90 s to make the sample surface flat and ensure a constant focal distance. Approximately 3 g of paraffin binder was placed under 3 mg of pyrotechnic delay to form pellets.

### Calorimetric method

#### DSC

Calorimetric analyses were performed using a commercial differential scanning calorimeter (Mettler Toledo Inc., Model DSC 3) in the temperature range 30–600 °C. Data on heat flow was collected in accordance with International Confederation for Thermal Analyses and Calorimetry standards. Experiments were carried out under a nitrogen flow (80 mL/min). Each sample was prepared with 2 mg of powder in a 40 μm diameter placed in a standard pierced aluminium pan. A DSC curve for each ageing condition was constructed by averaging the data from three replicate experiments. A high level of reliability was secured for temperature accuracy (±0.2 K), temperature precision (±0.02 K) and calorimetric accuracy (±2%).

## Supplementary information

Supplementary Information
