# Supplementary material for: A non-calorimetric approach for investigating the moisture-induced ageing of a pyrotechnic delay material using spectroscopies
Source: Sci Rep. 2019 Oct 23;9:15228. doi: 10.1038/s41598-019-51667-y (PMC6811628; doi:10.1038/s41598-019-51667-y)
Supplement: Supplementary file 1 — Supplementary Information [file 41598_2019_51667_MOESM1_ESM.pdf]

Electronic Supplementary Information

For

A non-calorimetric approach for investigating the moisture-induced ageing of a pyrotechnic delay material using spectroscopies

Ji-Hoon Ryu, Jun-Ho Yang, and Jack J. Yoh*

Department of Mechanical and Aerospace Engineering, Seoul National University, Seoul, 08826, South Korea

* Corresponding author: *jjyoh@snu.ac.kr*

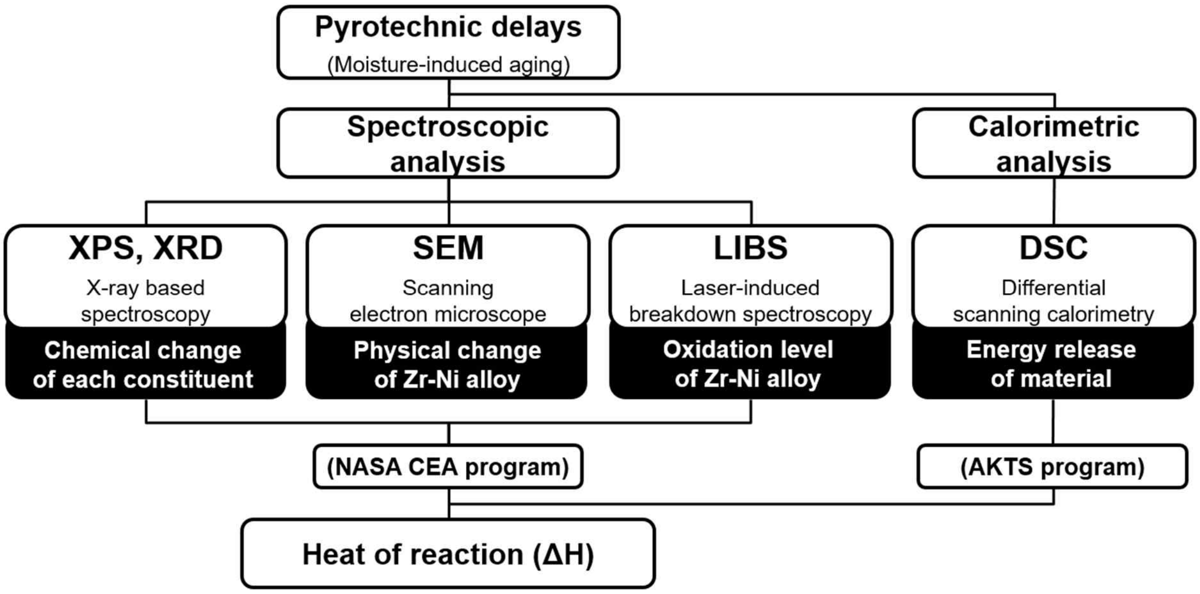

Figure S1. The detailed process of the non-calorimetric and calorimetric analysis.

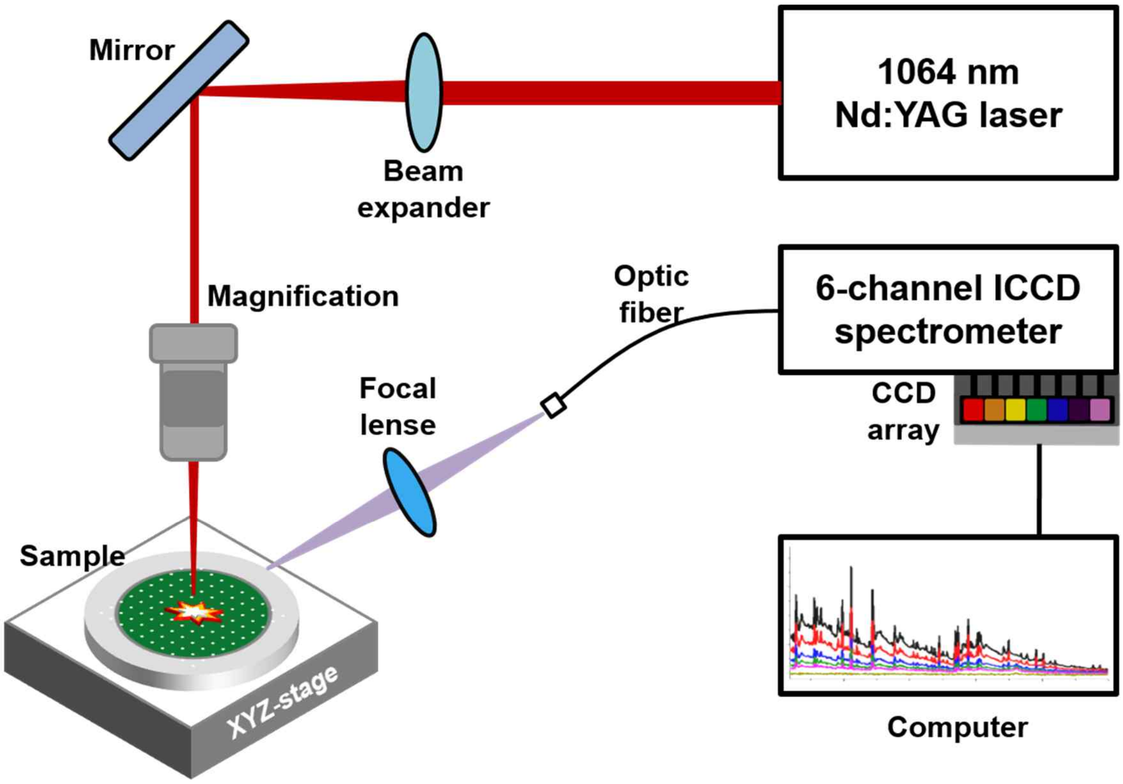

Figure S2. An overall schematic of the set up used for LIBS analysis.

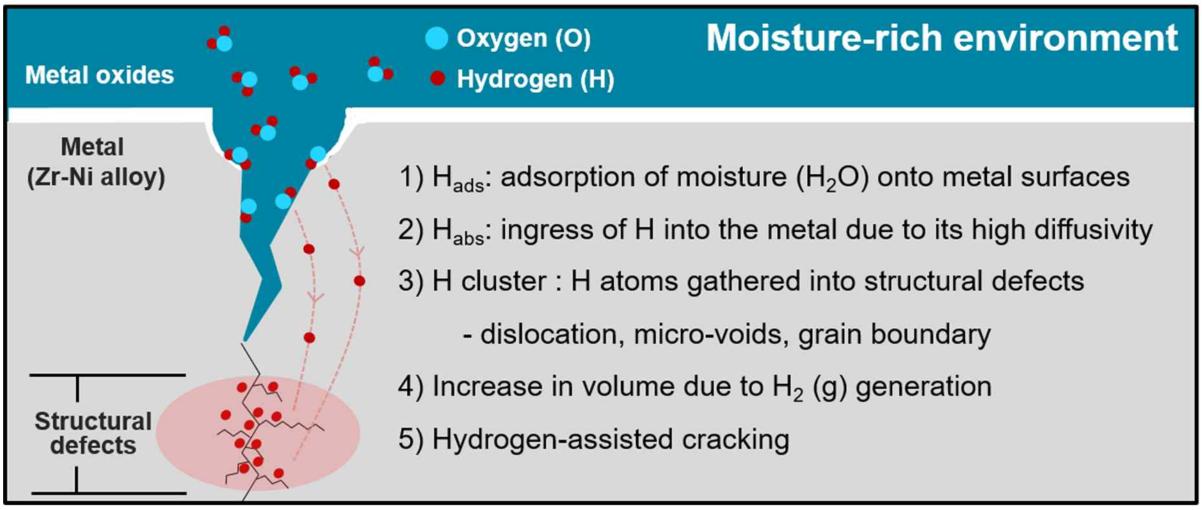

Figure S3. A detailed description of the hydrogen-assisted cracking process on metals under excessive moisture conditions.

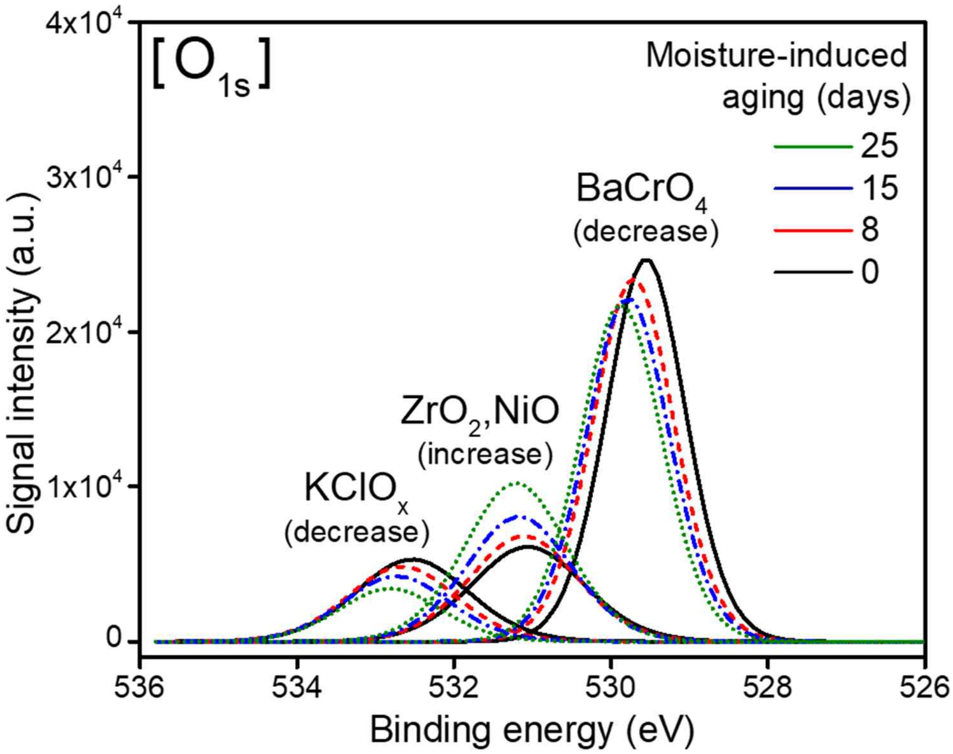

Figure S4. O1s XPS peaks for a pyrotechnic delay material at different levels of moisture-induced ageing. The signal for $BaCrO_4$ (529.40 eV), NiO (531.00 eV), ZrO_2 (531.17 eV), $KClO_3$ (532.60 eV), and $KClO_4$ (532.70 eV) gradually changes with ageing.
